# A multi-omics analysis of pancreatitis: bridging familial genetics and disease progression

**DOI:** 10.3389/fimmu.2025.1707821

**Published:** 2026-01-12

**Authors:** Fu Li, Jin-xin Huang, Wen-jie Sun, Jing-qing Zeng, Ke-xin Gan, Biao Gong, Jian-mei Ji, Jian Chen, Zhao-hui Deng, Dong-liang Xu

**Affiliations:** 1 Department of Hepatobiliary Pancreatic Surgery, Shuguang Hospital Affiliated to Shanghai University of Traditional Chinese Medicine, Shanghai, China; 2 Traditional Chinese Medicine Integrated Department of Nephrology, The First Affiliated Hospital of Zhengzhou University, Zhengzhou, China; 3 Department of Gastroenterology, Shanghai Children’s Medical Center, School of Medicine, Shanghai Jiao Tong University, Shanghai, China; 4 Department of Gastroenterology, Shuguang Hospital Affiliated to Shanghai University of Traditional Chinese Medicine, Shanghai, China; 5 Digestive Endoscopy Center of Shuguang Hospital Affiliated to Shanghai University of Traditional Chinese Medicine, Shanghai, China; 6 Institute of Vascular Anomalies, Shanghai TCM-Integrated Hospital, Shanghai University of Traditional Chinese Medicine, Shanghai, China; 7 Department of Public Health, International College, Krirk University, Bangkok, Thailand; 8 Anhui Province Rural Revitalization Collaborative Technical Service Center, Huangshan University, Huangshan, China

**Keywords:** acute pancreatitis, chronic pancreatitis, genetic mutation, immune microenvironment, machine learning

## Abstract

Chronic and acute pancreatitis (CP and AP, respectively) are debilitating conditions with significant morbidity and mortality, necessitating a comprehensive understanding of their underlying mechanisms. This study provides a high-resolution, multi-omics investigation into the genetic and immune cell underpinnings of pancreatitis, integrating rare familial CP with a large cohort of patients with AP. Utilizing an integrative approach that combined whole-exome sequencing (WES) from two pediatric CP patients and their family members with single-cell RNA sequencing (scRNA-seq) and bulk transcriptomics from a public AP cohort (*n* = 119), we identified a shared molecular and cellular pathology. WES of the CP family revealed heterozygous mutations in 12 novel genes, including *EXOC4*, *ATG2A*, and *UNC80*. Functional enrichment analysis highlighted autophagy, cell adhesion, and vesicle-mediated transport as the key biological processes implicated in the pathophysiology of both conditions. Single-cell profiling of peripheral blood mononuclear cells (PBMCs) from the CP family revealed a marked increase in the proportion of naive B cells and an altered activity of CD8^+^ T cells, suggesting a dysregulated B-cell-mediated immune response. This observation was corroborated in the AP cohort, where CIBERSORT analysis revealed a significant increase in both naive B cells and CD8^+^ T cells correlating with the disease severity. Weighted gene co-expression network analysis (WGCNA) on the AP cohort uncovered 14 gene modules associated with disease progression. These modules were significantly enriched for pathways central to the innate immune response, including complement-dependent cytotoxicity and neutrophil degranulation, providing a molecular link to the observed immune cell infiltration. An artificial intelligence (AI)-driven model incorporating 110 CP family-related genes (GTCPFs) demonstrated exceptional predictive capability (average AUC > 0.84) for AP severity, highlighting the translational potential of our findings. The model identified a robust signature of 17 genes, including *ATG2A*, *EXOC4*, and *TNS1*, which may serve as novel diagnostic and prognostic biomarkers. Our findings provide a unified view of the pathogenesis of pancreatitis, linking novel genetic variants to specific immune cell and transcriptomic signatures. This integrative approach underscores the critical importance of both genetic and immune factors in CP and AP, identifying potential biomarkers and therapeutic targets and paving the way for personalized medicine in the management of these challenging conditions.

## Graphical Abstract

**Figure:**
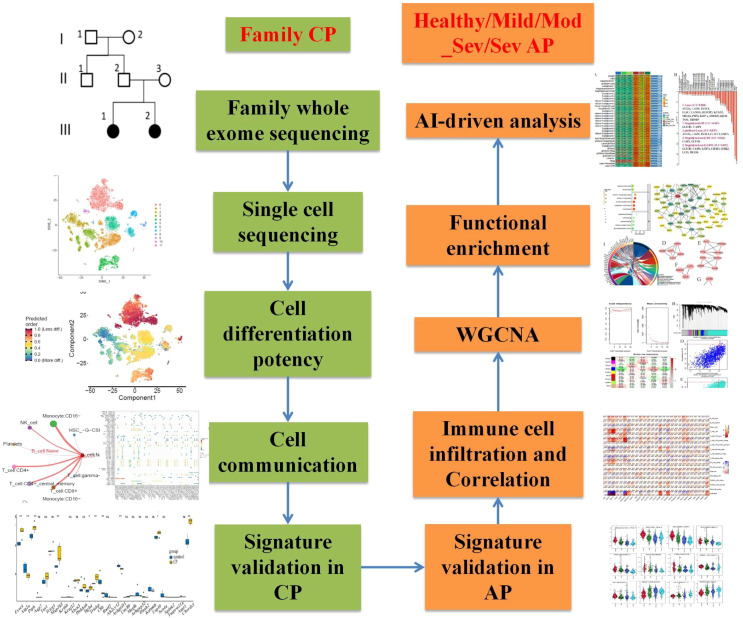

## Introduction

Chronic pancreatitis (CP) is a debilitating digestive disorder characterized by persistent inflammation of the pancreas, leading to significant clinical manifestations such as abdominal pain, malabsorption, and nutritional deficiencies (1). The current management strategies primarily involve pharmacological treatment, dietary modifications, and surgical interventions. However, these approaches often fall short in terms of symptom alleviation and disease management, particularly with regard to etiological investigations and personalized treatment options (2). Acute pancreatitis (AP) and CP share overlapping biological processes but are typically studied in isolation. This separation has limited our ability to translate the findings from rare familial forms to common sporadic presentations. Thus, exploration of the pathogenic mechanisms that connect familial genetic predisposition to the immune microenvironmental changes in both CP and AP is critical to the development of innovative diagnostic and therapeutic strategies that can improve patient outcomes.

There is a notable research gap: although individual genetic variants (e.g., *PRSS1* and *SPINK1*) have been implicated in hereditary pancreatitis, and immune dysregulation has been observed in pancreatitis, there is a lack of studies that systematically interrogate how rare, highly penetrant genetic variants influence the immune cell states and intercellular communication—and whether these effects recapitulate in sporadic AP (3). Familial CP therefore provides a unique window to discovering causal genetic perturbations whose downstream transcriptional and cellular consequences can be traced and examined in larger AP cohorts (4). We hypothesize that the genetic predispositions identified in rare familial CP cases will illuminate shared molecular and cellular pathways—particularly those related to autophagy, vesicle trafficking, and immune cell interactions—that are also dysregulated across the spectrum of AP severity.

To test this unified hypothesis, we adopted a deliberate multi-omics strategy: whole-exome sequencing (WES) to discover rare, segregating variants in a multiplex family with early-onset CP; single-cell RNA sequencing (scRNA-seq) of peripheral blood mononuclear cells (PBMCs) to resolve cell type-specific transcriptional changes and ligand–receptor interactions; and bulk transcriptome analysis of well-phenotyped AP cohorts to validate and generalize family-level discoveries at the population scale (5). WES is ideally suited to the detection of novel coding variants with Mendelian segregation, but it does not reveal the cellular contexts through which such variants exert pathophysiological effects (6–8). scRNA-seq provides the single-cell resolution necessary to link the genotype to the altered immune cell composition, differentiation states, and intercellular signaling, while bulk cohort analysis allows assessment of whether the same gene expression modules and pathway perturbations are associated with the severity of AP in independent samples (9, 10).

Moreover, prior studies have implicated both the adaptive and innate immune components in the pathogenesis of pancreatitis: for example, the roles for the B-cell subsets and T-cell dysfunction have been described; however, these findings have not been comprehensively integrated with genetic data (11, 12). Building on this literature, our approach specifically interrogates whether candidate genetic variants correlate with the shifts in the B-cell and T-cell populations, the altered CD8^+^ T-cell states, and the innate immune effectors (e.g., neutrophil degranulation and complement activity), thereby connecting molecular genetics to immune mechanisms (9, 13).

In summary, this study addresses a clear and actionable gap by combining family-based WES, scRNA-seq of PBMCs, and bulk transcriptomic validation in order to i) identify candidate pathogenic variants in familial CP; ii) map their impacts on the immune cell composition and intercellular communication at single-cell resolution; and iii) determine whether these molecular signatures are conserved and prognostically relevant across AP severities. By explicitly linking rare variant discovery to immune reprogramming and population-level validation, our aim was to provide a unified mechanistic framework that can guide targeted experimental validation and ultimately inform precision diagnostics and therapeutics for pancreatitis.

## Materials and methods

### Clinical information

The current research was granted ethical clearance by the local Institutional Review Board of Shuguang Hospital affiliated with Shanghai University of Traditional Chinese Medicine (approval no. 2023-1356-123-01). Our investigation encompassed two sisters diagnosed with CP who had been under the care of our hospital, along with five members of their familial lineage. Written informed consent was obtained from the parents/guardians of pediatric participants, and all samples were de-identified and stored under restricted access.

### Whole-exome sequencing

In the present study, genomic DNA was extracted from the peripheral blood samples of two affected siblings (III-1, 3 years old; III-2, 5 years old) diagnosed with CP and five unaffected relatives according to the protocol provided by Tiangen Biotech (Beijing, China). Subsequently, the quantity and integrity of the extracted DNA were assessed using a NanoDrop spectrophotometer (Thermo Fisher Scientific, Inc., Wilmington, DE, USA) and 1% agarose gel electrophoresis, respectively. To obtain genomic DNA samples from seven family members, the Agilent SureSelect Human All Exon v6 library was utilized in accordance with the manufacturer’s instructions (Agilent Technologies, Santa Clara, CA, USA). This procedure entailed shearing approximately 130 μl (3 μg) of genomic DNA into fragments ranging from 150 to 220 bp using a sonicator (Covaris, Inc., Woburn, MA, USA). The fragmented DNA was then purified and subjected to end-polishing, followed by the ligation of adapters provided by Agilent. The resultant libraries were amplified through polymerase chain reaction (PCR) and hybridized with customized probes. The probe-bound DNA fragments were thoroughly washed and eluted using the buffer included in the kit. The prepared libraries were then sequenced on the Illumina HiSeq X-10 platform (Illumina, Inc., San Diego, CA, USA), yielding 150 bp paired-end reads. The entire exome sequencing and subsequent data analysis were professionally conducted by OE Biotech Co., Ltd. (Shanghai, China).

### Bioinformatics analysis of the whole-exome sequencing

The raw sequencing data were initially organized in the fastq format and subsequently subjected to rigorous preprocessing with fastp (v0.19.5) (14), the aim of which was to filter out low-quality sequences and ensure the integrity of the reads for downstream analyses. This preprocessing entailed trimming adapter sequences, removing bases with average quality scores below 20 within sliding windows, and discarding reads containing ambiguous nucleotides or those shorter than 75 bp. The resulting clean reads were then aligned to the reference human genome (GRCh37) using BWA (v0.7.12) (15), and the aligned reads were further sorted and indexed with SAMtools (v1.4) (16). To enhance the accuracy of variant calling, the base quality scores were recalibrated and the single nucleotide polymorphisms (SNPs) and insertion/deletions (indels) were realigned using GATK (v4.1.0.0) (17), with duplicate reads marked by Picard (v4.1.0.0). These steps yielded BAM files ready for variant analysis. During SNP and indel calling, various annotation databases including RefSeq, 1000 Genomes, COSMIC, and OMIM were consulted, and ANNOVAR [v(unspecified)] (18) was utilized for annotation purposes. Furthermore, CNVkit (v0.9.5) (19) was employed to infer the copy number variations (CNVs) from the sequencing data, while Lumpy (v0.2.13) (20) was utilized for the detection of structural variations (SVs). The identified genomic variations were subsequently visualized in a Circos diagram, providing a comprehensive and intuitive representation of the genomic alterations.

### Screening of candidate SNPs/indels for CP

In the initial step, we excluded all mutations that exhibited a frequency exceeding 1% in any of the 1000g2015aug_eas database. For the purpose of refining our mutation set, we implemented a dual-pronged approach. Firstly, we retained the mutations that were deemed potentially harmful by 12 predictive software tools: SIFT (21), Polyphen2 HDIV (22), Polyphen2_HVAR (22), LRT (23), MutationTaster (24), MutationAssessor (25), FATHMM (26), fathmm-MKL_coding (27), PROVEAN (28), MetaSVM (29), MetaLR (29), and CADD (30). Secondly, we included the mutations found exclusively in patients with CP, which were absent in healthy controls. Consequently, we selected the mutations classified as pathogenic or likely pathogenic as potential candidate sites. In addition, dominant genetic pattern was performed to obtain the dominant genetic pattern mutation sites. These sites were subsequently designated as candidate sites for further analysis.

### Functional enrichment and PPI analyses

To elucidate the functional roles of the SNP/indel-associated candidate genes, we performed comprehensive Gene Ontology (GO) enrichment analyses, encompassing the biological process (BP), molecular function (MF), and cellular component (CC) categories, alongside pathway analysis incorporating the Reactome, Kyoto Encyclopedia of Genes and Genomes (KEGG), WikiPathways, and BioCarta databases, utilizing the Metascape platform (31). In addition, we conducted a protein–protein interaction (PPI) network analysis for these candidate genes through the STRING database (https://cn.string-db.org). The Molecular Complex Detection (MCODE) algorithm (32) was employed to pinpoint the highly interlinked network segments within the dataset.

### Single-cell RNA-seq library preparation and sequencing

Human blood cells from four individuals, namely the elder sister, the younger sister, the father, and the mother, were subjected to scRNA-seq. For the preparation and sequencing of the scRNA-seq libraries, the cells were loaded onto the microfluidic chip of the Chip A Single Cell Kit v2.1 [catalog no. S050100301; MobiDrop (Zhejiang) Co., Ltd., Tongxiang, China]. This process enabled the formation of droplets containing MobiNova-100 [catalog no. A1A40001; MobiDrop (Zhejiang) Co., Ltd.], wherein each cell was encapsulated within a droplet along with a gel bead linked to millions of oligos bearing unique cell barcodes. After encapsulation, the droplets were optically cut using the MobiNovaSP-100 [catalog no. A2A40001; MobiDrop (Zhejiang) Co., Ltd.], facilitating the diffusion of the oligos into the reaction mixture. Inside these droplets, the messenger RNAs (mRNAs) were captured by the cell barcodes and underwent complementary DNA (cDNA) amplification. Subsequent to reverse transcription, the resultant barcoded cDNAs were amplified, and a library was constructed using the High Throughput Single-Cell 3′ Transcriptome Kit v2.1 [catalog no. S050200301; MobiDrop (Zhejiang) Co., Ltd.] in combination with the 3′ Dual Index Kit [catalog no. S050300301; MobiDrop (Zhejiang) Co., Ltd.] (33, 34). Finally, the prepared libraries were sequenced on an Illumina NovaSeq 6000 System.

### Single-cell 3′ transcriptome data process

The raw data in fastq format obtained from the single-cell 3′ transcriptome sequencing were subjected to preliminary analysis via the MobiVision software (version 3.0, MobiDrop). The reads were mapped to the *Homo sapiens* reference genome GRCh38. By means of MobiVision, a filtered cell–gene matrix was produced, with low-quality cells being excluded in accordance with established data curation methods (34). This refined dataset served as the foundation for subsequent analytical endeavors.

### Single-cell RNA-seq data preprocessing

Following rigorous data preprocessing, which involved the exclusion of cells according to criteria such as having fewer than 200 genes, more than 5,000 genes, or a mitochondrial gene content surpassing 25%, a refined dataset comprising 13,690 cells was obtained for analysis. Batch effects in the scRNA-seq dataset were corrected using the Harmony integration algorithm, which was applied after principal component analysis (PCA) to remove inter-sample technical variations while preserving the biological structure (35). Subsequent to this, we utilized the *t*-distributed stochastic neighbor embedding (t-SNE) technique for data visualization. Afterward, the FindNeighbors and FindClusters functions (with a resolution of 0.1) were employed to identify discrete cell clusters. For the annotation of these clusters, the singleR package was employed. Moreover, the CytoTRACE package (version 0.3.3) was applied to predict the sequential advancement of the cell differentiation and stemness states (36). To obtain an in-depth understanding of intercellular communication, a comprehensive analysis was carried out utilizing the CellChat 1.0.0 package. A ridge plot was employed to present the CytoTRACE scores of the diverse cell types in the control and CP groups. A bubble map was utilized to depict the expression of the hub genes in the different cell types. The GSE41418 dataset, which included pancreatic samples from six cerulein-induced CP mice and six healthy mice, was retrieved from the Gene Expression Omnibus (GEO) database (https://ncbi.nlm.nih.gov/geo) to validate the aforementioned results.

### Profile of the immune cell infiltration

To evaluate the significance of the immune microenvironment in AP progression, we leveraged the Sangerbox 3.0 platform (37) to scrutinize and contrast the patterns of the immune cell infiltration between the AP cohorts and healthy controls through the CIBERSORT algorithm. This tool is specifically designed for the accurate quantification of the relative abundances of 22 immune cell subsets within complex gene expression profiles. Subsequently, Pearson’s correlation analysis was performed to evaluate the relationship between the CP family-related genes (GTCPFs) and the infiltrating immune cells.

### Weighted gene co-expression network analysis

To unveil the tightly interconnected gene clusters and key hub genes, the weighted gene co-expression network analysis (WGCNA) methodology was employed to construct scale-free gene co-expression networks from the GSE194331 dataset, which included 57 cases of mild AP, 20 cases of moderately severe AP, 10 cases of severe AP, and 32 control subjects. For the gene set variation analysis (GSVA) of the previously defined GTCPF gene set, Sangerbox 3.0 was utilized to calculate the gene enrichment score of each sample.

The analytical procedure was initiated with the application of hierarchical clustering based on Pearson’s correlations to effectively classify all genes and samples. Subsequently, the optimal soft threshold power value was determined, which is essential for the establishment of a co-expression network conforming to the principles of scale-free topology. The adjacency matrices obtained from this process were then transformed into topological overlap matrices, which facilitated the identification of distinct gene modules. The analysis was further refined by merging similar modules and clustering them accordingly. Finally, module–trait relationship diagrams were generated to illustrate the associations between gene modules and traits, and a comprehensive inventory of the genes relevant to each module was compiled.

### Signature obtained from artificial intelligence-driven integrative approaches

To guarantee a precise and reliable consensus artificial intelligence-driven signature associated with GTCPFs, 10 machine learning (ML) algorithms were integrated and 113 algorithmic permutations were evaluated. These combined methods included Cox, CoxBoost, elastic net (Enet), generalized boosted regression modeling (GBM), LASSO (least absolute shrinkage and selection operator), plsRcox for Cox, random survival forest (RSF), Ridge, supervised principal components (SuperPC), and survival support vector machine (survival-SVM). The development procedures were as follows:

a) The 113 algorithmic permutations were applied to the GTCPF-related signature using the training cohort (GSE194331, healthy *vs*. AP).

b) Subsequently, these models were validated with five testing cohorts (healthy *vs*. Mild AP, healthy *vs*. Moderate_Severe AP, Mild AP *vs*. Moderate_Severe AP, Mild AP *vs*. Severe AP, and Moderate_Severe AP *vs*. Severe AP).

c) For each model, the area under the receiver operating characteristic curve (AUC) was calculated across all datasets.

### Screening of potential diagnostic biomarkers for AP/SAP

To identify the severity-associated GTCPF biomarkers in AP, we determined the expression of the 29 GTCPF genes from the GSE194331 cohort after background correction, log2 transformation, and quantile normalization. The samples were categorized into the control, mild, moderately severe, and severe AP groups. One-way ANOVA followed by Benjamini–Hochberg correction was applied to compare the gene expression across the four severity groups. Genes with a false discovery rate (FDR) <0.05 were considered significantly severity-associated.

### Statistical analysis

All statistics were run in R 4.0.5. Values are quoted as the mean ± SEM. Pairwise comparisons were performed with an unpaired *t*-test, and three or more groups were analyzed using one-way ANOVA. The level of statistical significance was set at *p* < 0.05.

## Results

### Clinical information and mutational landscape

**Figure 1:**
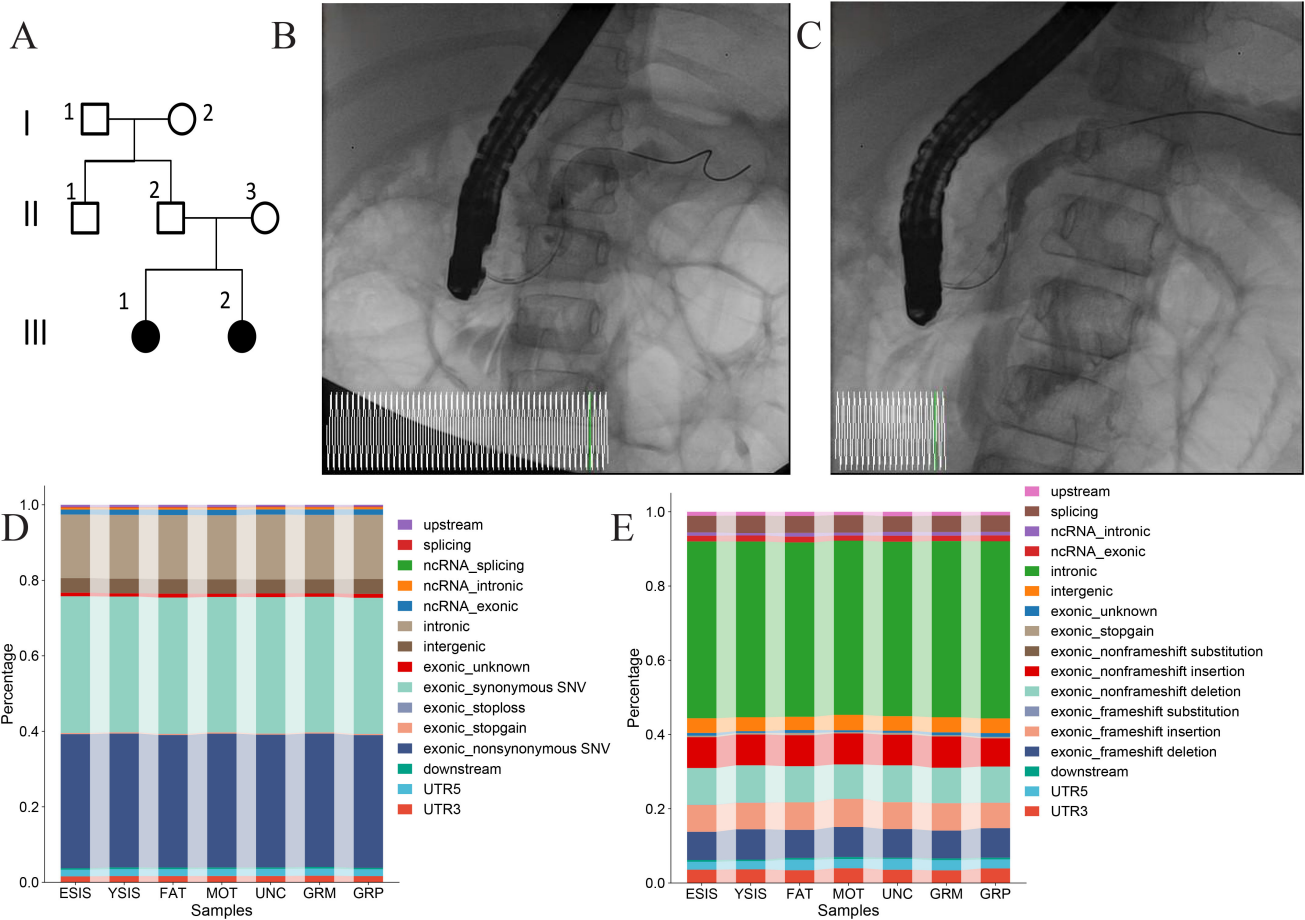
Pedigree plot of chronic pancreatitis and the distribution of mutations. **(A)** Pedigree plot of chronic pancreatitis. **(B, C)** Endoscopic retrograde cholangiopancreatography of the two patients. **(D, E)** Distribution across genomic features of all single-nucleotide variations (SNVs) **(D)** and insertion/deletions (indels) **(E)**.

The pedigree of the studied family is shown in **Figure 1A**, with two affected siblings (III-1, 3 years old; III-2, 5 years old) diagnosed with CP and five unaffected relatives. Both affected children underwent endoscopic retrograde cholangiopancreatography to confirm the disease (**Figures 1B, C**). Biochemical evidence included a markedly elevated serum amylase (peak 1,300 U/L in III-1 and >600 U/L in III-2). The younger sibling had a disease duration of approximately 1 year and met the Cambridge criteria for severe CP. The older sibling had a disease duration of approximately 1 year and 2 months and was classified as having moderate CP according to the Cambridge grading system. Across the seven WES samples, the total single nucleotide variant counts ranged from 18,392 to 18,741, with variants predominantly mapping to the exonic, intronic, and intergenic regions (**Figure 1D**). The indel counts per sample ranged from 1,896 to 1,951 and were mainly localized to the intronic and exonic regions (**Figure 1E**).

### Candidate mutations segregating with familial CP

Using a dominant inheritance model and multi-algorithm pathogenicity filtering, we prioritized 12 heterozygous genes that segregated uniquely with the disease in the affected family: *EXOC4*, *ATG2A*, *UNC80*, *USP10*, *ARHGAP32*, *DNAH2*, *BATF2*, *SPINK2*, *AMZ1*, *MAP3K5*, *KNCN*, and *KDM6B*. These variants were absent in the five unaffected relatives, supporting co-segregation with the CP phenotype (**Figure 1A**; **Table 1**). Notably, the majority of these genes had not been previously associated with CP, suggesting novel candidate loci for early-onset familial disease. To capture additional potentially relevant loci, we also compiled a secondary list of 17 genes showing high-impact or severity-associated mutations (i.e., *MEGF6*, *C8B*, *TCIRG1*, *KCNJ12*, *SCN4A*, *ITGB4*, *PSPH*, *TMPRSS11A*, *ARHGEF11*, *TNS1*, *AQP7*, *ABCA3*, *KRT6B*, *PLEKHA6*, *FLACC1*, *CHORDC1*, and *TRIOBP*). Functional consequence assessment indicated that each of the 29 prioritized genes harbored at least one protein-altering variant (non-synonymous, frameshift, or in-frame indel), providing preliminary evidence for potential functional impact in CP (see **Table 1**).

**Table 1:** Protein changes of 29 genes.

Gene name	Region	Function	AA change	SNP	Screening method
*EXOC4*	Intronic			rs143105018	Dominant inheritance pattern
*ATG2A*	Exonic	Non-synonymous	NM_001367971.1:exon14:c.C1880T:p.A627V	rs2285347	Dominant inheritance pattern
*UNC80*	Exonic	Synonymous	NM_182587.4:exon63:c.G9675A:p.T3225T	rs141603698	Dominant inheritance pattern
*USP10*	Intronic			rs2641688	Dominant inheritance pattern
				rs3213810	
*ARHGAP32*	Exonic	Synonymous	NM_014715.4:exon13:c.A4863G:p.E1621E	rs3740829	Dominant inheritance pattern
		Non-synonymous	NM_014715.4:exon13:c.G4493C:p.S1498T	rs60847789	
*DNAH2*	Exonic	Non-synonymous	NM_020877.5:exon38:c.C5984T:p.P1995L	rs75886914	Dominant inheritance pattern
*BATF2*	UTR3			rs56759307	Dominant inheritance pattern
*SPINK2*	Exonic	Non-synonymous	NM_001271718.2:exon1:c.A118G:p.T40A	rs781543	Dominant inheritance pattern
			NM_001271718.2:exon1:c.G110A:p.G37E	rs781544	
*AMZ1*	Exonic	Non-synonymous	NM_001384739.1:exon7:c.G1403A:p.R468H	rs7776970	Dominant inheritance pattern
*MAP3K5*	Intronic			rs2277100	Dominant inheritance pattern
*KNCN*	Intronic	Indel		rs60803470	Dominant inheritance pattern
				rs376333496	
*KDM6B*	Exonic	Non-frameshift deletion	NM_001080424.2:exon11:c.2256_2261del:p.752_754del		Dominant inheritance pattern
*MEGF6*	Exonic	Non-synonymous	NM_001409.4:exon8:c.C964T:p.R322W	rs57542881	Harmful screening
*C8B*	Exonic	Non-synonymous	NM_000066.4:exon9:c.G1355C:p.W452S	rs200077558	Harmful screening
*TCIRG1*	Exonic	Non-synonymous	NM_006019.4:exon6:c.C629T:p.T210M	rs372826788	Harmful screening
*KCNJ12*	Exonic	Non-synonymous	NM_021012.5:exon3:c.G782A:p.R261H	rs77270326	Harmful screening
			NM_021012.5:exon3:c.G415A:p.E139K	rs76265595	
			NM_021012.5:exon3:c.G433A:p.G145S	rs75029097	
			NM_021012.5:exon3:c.T785G:p.I262S	rs76684759	
			NM_021012.5:exon3:c.C425A:p.T142N	rs76518282	
*SCN4A*	Exonic	Non-synonymous	NM_000334.4:exon24:c.G4705A:p.G1569S	rs774821803	Harmful screening
*ITGB4*	Exonic	Non-synonymous	NM_001005619.1:exon6:c.C599G:p.P200R		Harmful screening
*PSPH*	Exonic	Non-synonymous	NM_001370509.1:exon4:c.G194A:p.R65H	rs200442078	Harmful screening
			NM_001370509.1:exon4:c.G268A:p.G90S	rs75395437	
*TMPRSS11A*	Exonic	Non-synonymous	NM_001114387.2:exon8:c.T932G:p.F311C		Harmful screening
*ARHGEF11*	Exonic	Non-synonymous	NM_014784.4:exon13:c.T1027C:p.Y343H		Harmful screening
*TNS1*	Exonic	Non-synonymous	NM_001308022.2:exon16:c.C1486T:p.R496W	rs758441152	Harmful screening
*AQP7*	Exonic	Non-synonymous	NM_001318156.2:exon6:c.T521C:p.L174P	rs145516206	Harmful screening
			NM_001318156.2:exon4:c.T172C:p.Y58H	rs74668961	
*ABCA3*	Exonic	Non-synonymous	NM_001089.3:exon16:c.C1960G:p.L654V	rs150910102	Harmful screening
*KRT6B*	Exonic	Non-synonymous	NM_005555.4:exon1:c.G332A:p.G111D	rs61745883	Harmful screening
*PLEKHA6*	Intronic	Indel			Harmful screening
*FLACC1*	Intronic	Indel		rs571747757	Harmful screening
*CHORDC1*	Intronic	Indel		rs535554925	Harmful screening
*TRIOBP*	Exonic	Non-frameshift deletion	NM_001039141.3:exon7:c.1192_1194del:p.398_398del	rs55745992	Harmful screening

### Significantly enriched GO and pathways terms of potential CP-related genes

**Figure 2:**
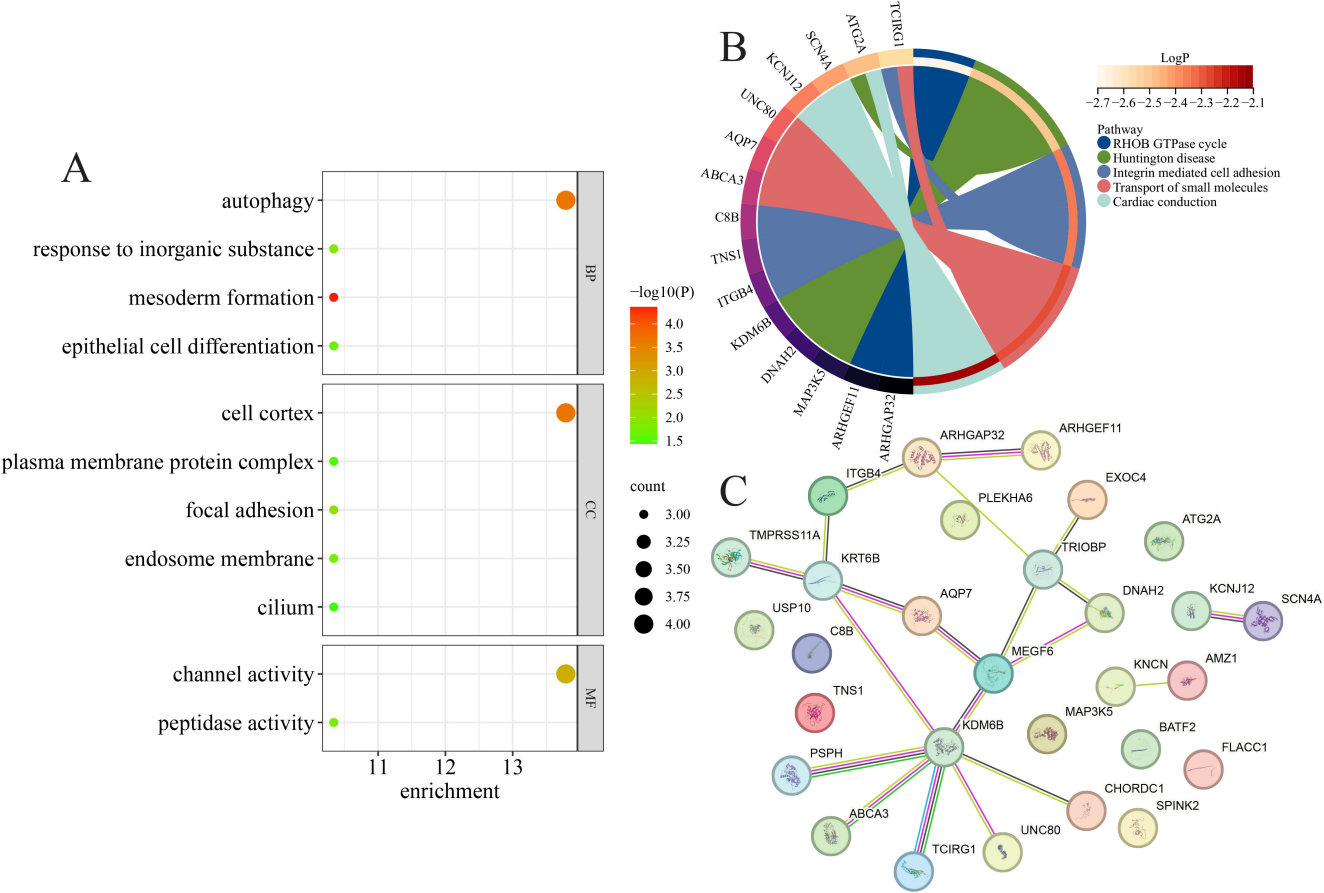
Significantly enriched functions and protein–protein interaction (PPI) network of the 29 chronic pancreatitis (CP)-related genes. **(A, B)** Significantly enriched biological processes, cellular components, and molecular functions **(A)** and pathways **(B)**. **(C)** PPI network of the 29 CP-related genes.

We performed GO (BP/CC/MF) and pathway enrichment analyses on the 29 candidate genes to infer shared biological themes (**Figure 2**). The biological processes most strongly enriched included autophagy, epithelial cell differentiation, and response to inorganic substances, while the enriched cellular components comprised the cell cortex, focal adhesion, and endosome membrane. At the molecular function level, channel activity and peptidase activity were overrepresented (**Figure 2A**). Pathway enrichment highlighted RHOB GTPase cycle, integrin-mediated cell adhesion, and transport of small molecules as recurrent pathways, with the hub genes such as *TCIRG1*, *ATG2A*, *SCN4A*, *UNC80*, and *AQP7* featuring prominently (**Figure 2B**). PPI mapping revealed that the majority of the candidates were isolated nodes, but a small subset (including *KDM6B*, *KRT6B*, *MEGF6*, *ARHGAP32*, and *TRIOBP*) formed interconnected modules, suggesting focal sub-networks of functional relevance (**Figure 2C**).

### Analysis of the heterogeneity of PBMCs in the CP single-cell transcriptome

**Figure 3:**
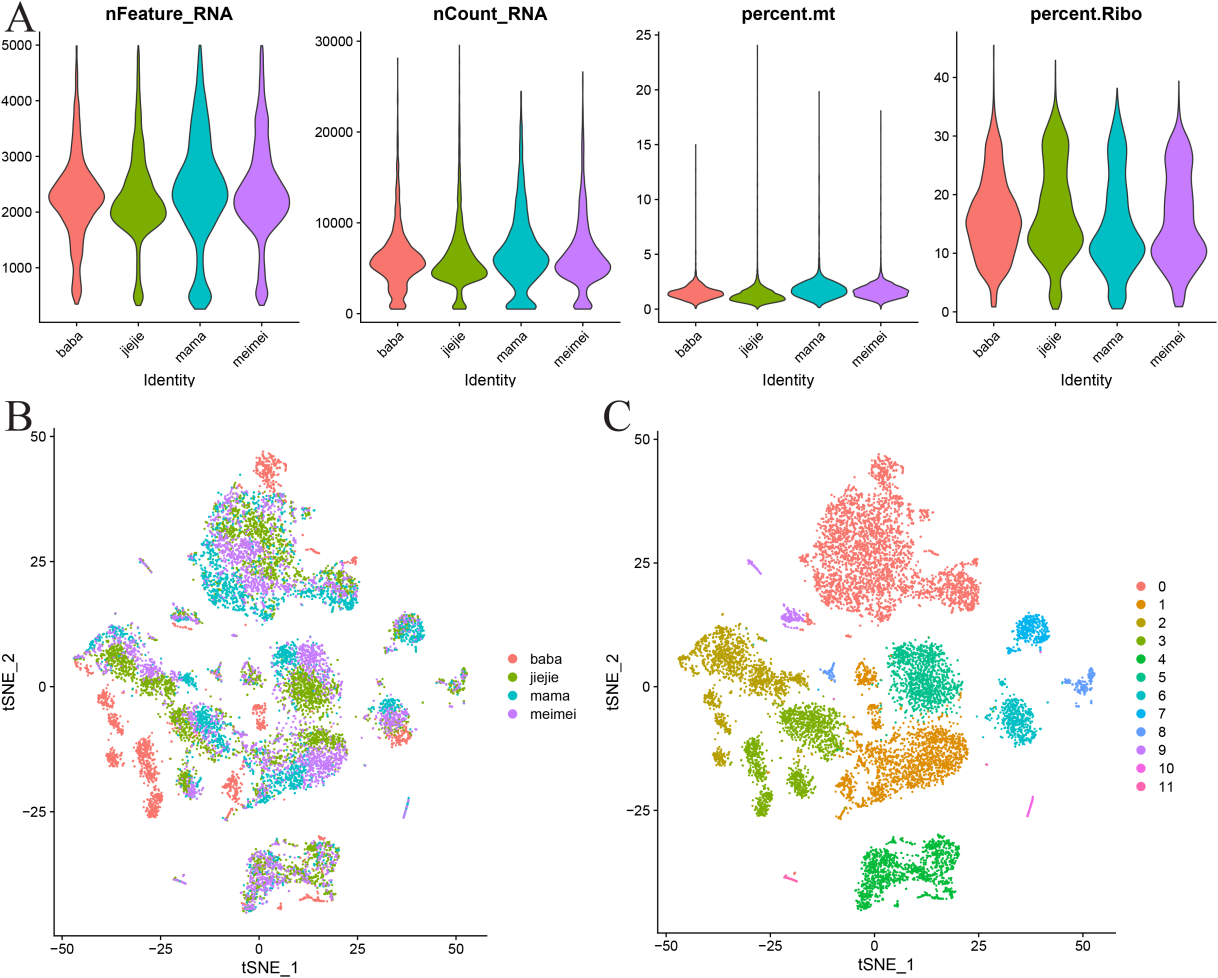
Optimization of the single-cell RNA sequencing (scRNA-seq) data for subsequent analytical procedures. **(A)** Initial data assessment and quality assurance of the mitochondrial and ribosome genes. **(B)***t*-distributed stochastic neighbor embedding (*t*-SNE) visualization of the harmonized datasets. **(C)** Identification of the cellular subpopulations through clustering with a resolution of 0.1.

**Figure 4:**
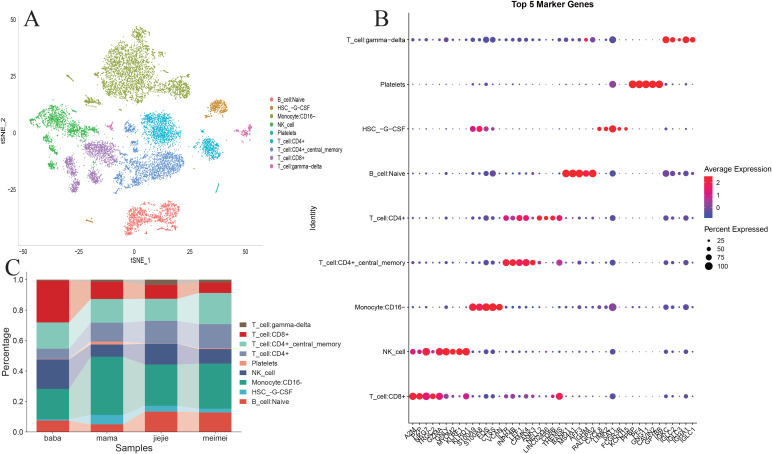
Analysis of the cellular proportions and marker gene expression in chronic pancreatitis (CP) *versus* control samples using single-cell RNA sequencing (scRNA-seq) data. **(A)***t*-Distributed stochastic neighbor embedding (*t*-SNE) visualization of different cell type distributions. **(B)** Heatmap depiction of the marker gene expression in the identified cells. **(C)** Comparison of the overall cell fractions.

We generated the scRNA-seq profiles from the PBMCs of two patients with CP (III1 and III2) and two healthy relatives (II2 and II3). After quality control and batch correction (Harmony), 13,690 cells were retained for downstream analysis (**Figures 3A, B**). Clustering (Seurat, resolution = 0.1) resolved 12 clusters that we annotated into nine major immune cell types using canonical markers: CD16^−^ monocytes (S100A9, S100A8, and FOS), CD4^+^ central memory T cells (IL7R, INPP4B, and LEF1), CD8^+^ T cells (A2M, PZP, and NKG7), natural killer (NK) cells (GZMA and GNLY), naive B cells (BANK1, MS4A1, and AFF3), CD4^+^ T cells (NELL2, CD8B, and THEMIS), hematopoietic stem cell granulocyte colony-stimulating factor (HSC-G-CSF) (CXCL8, LIMK2, and SAT1), γδ T cells (IGKC, IGLC2, and IGLC3), and platelets (PPBP, PF4, and GNG11) (**Figures 3C**, **4A, B**). The comparative cell composition analysis showed significant differences between CP and the controls, most notably an expansion of naive B cells and alterations in the CD8^+^ T-cell proportions in the CP samples (**Figure 4C**), implicating adaptive immune remodeling in familial CP.

### Cell differentiation potency analysis through CytoTRACE

**Figure 5:**
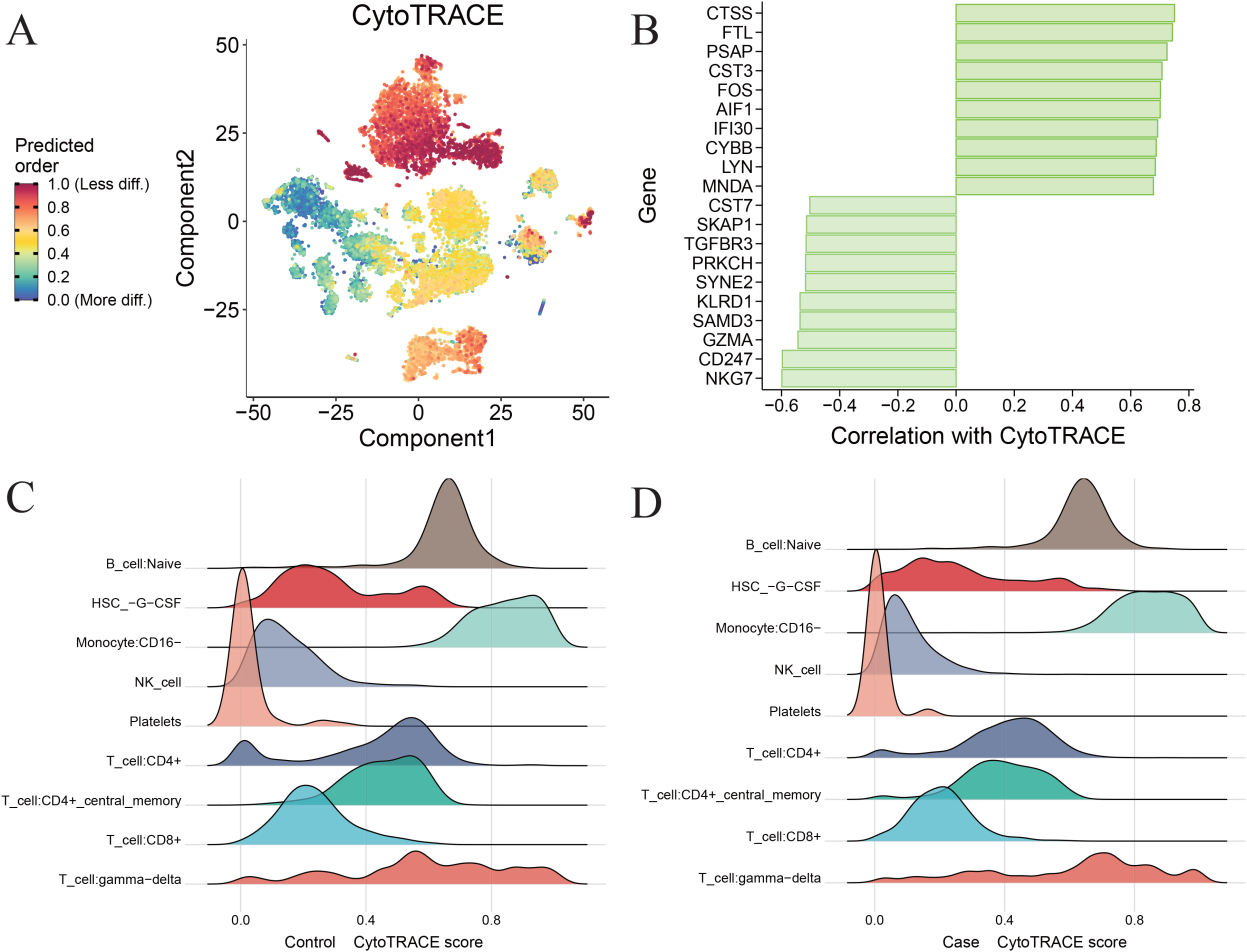
Trajectory analysis of all cells through CytoTRACE. **(A)***t*-Distributed stochastic neighbor embedding (*t*-SNE) visualization illustrating the level of stemness in the different cells predicted by CytoTRACE. **(B)** Correlation plot showing the genes that exhibit the strongest correlation with the CytoTRACE score. **(C, D)** Ridge plots showing the CytoTRACE scores of the different cells in the control **(C)** and chronic pancreatitis (CP) **(D)** groups.

To infer the differentiation states, we applied CytoTRACE across the annotated cell types. Platelets, NK cells, and CD8^+^ T cells scored as the most differentiated (lowest stemness), whereas CD16^−^ monocytes, γδ T cells, and naive B cells displayed the highest CytoTRACE scores (**Figure 5A**), indicating greater stemness or developmental potential. The genes most correlated with the CytoTRACE scores included *CTSS*, *FTL*, *PSAP*, *CST3*, *FOS*, *SAMD3*, *KLRD1*, *GZMA*, *CD247*, and *NKG7* (**Figure 5B**). Compared with the controls, the CP samples showed significant shifts in the T-cell differentiation metrics: the γδ T-cell scores were increased, while the CD4^+^ central memory and CD4^+^ T-cell scores were decreased. The CD16^−^ monocyte stemness was also reduced in CP (**Figures 5C, D**). These results suggest altered differentiation trajectories of both the innate and adaptive compartments in CP.

### CellChat identified communication patterns in the CP-PBMC microenvironment

**Figure 6:**
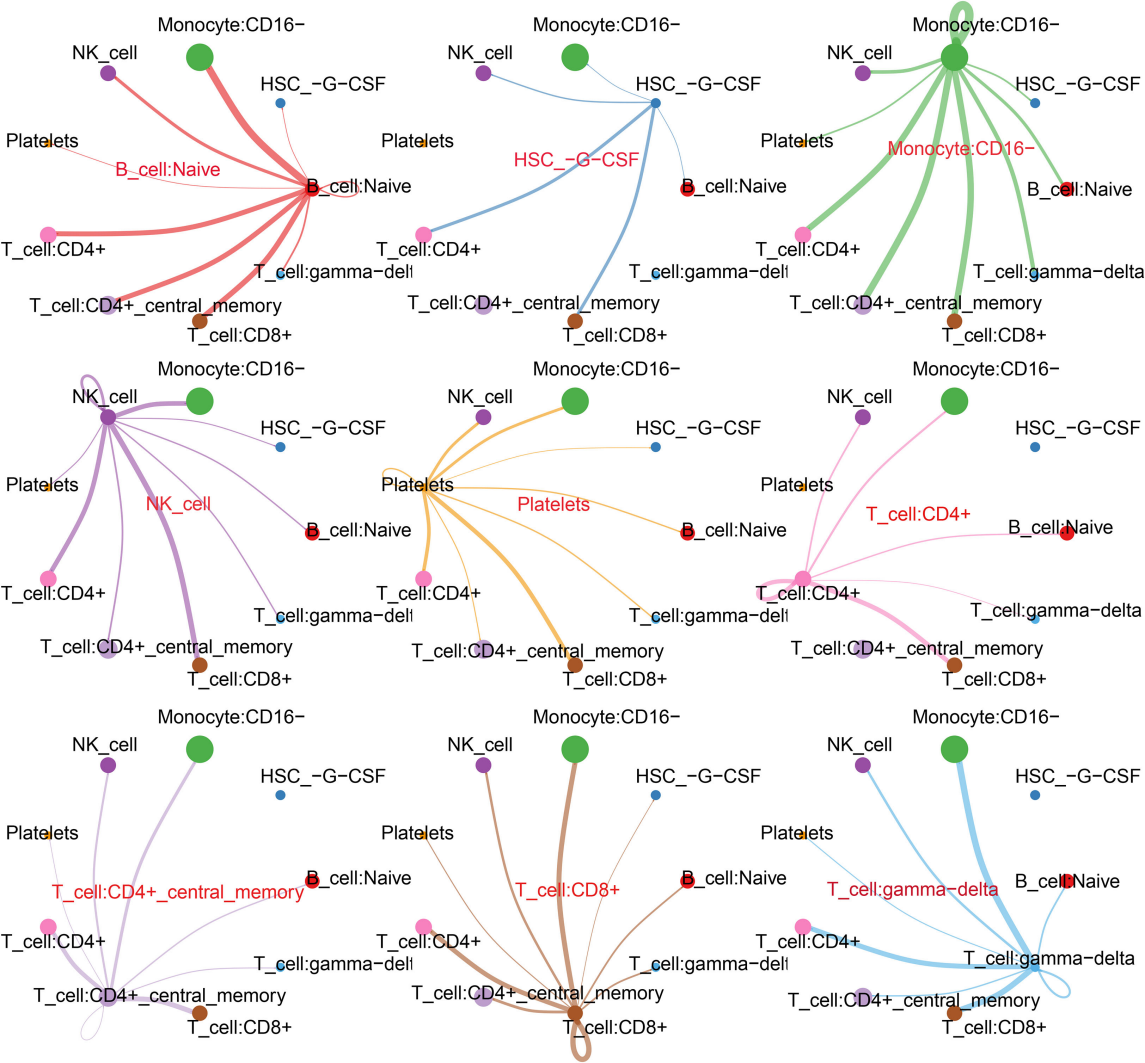
Overview of the CellChat communications in the chronic pancreatitis peripheral blood mononuclear cell (CP-PBMC) microenvironment.

**Figure 7:**
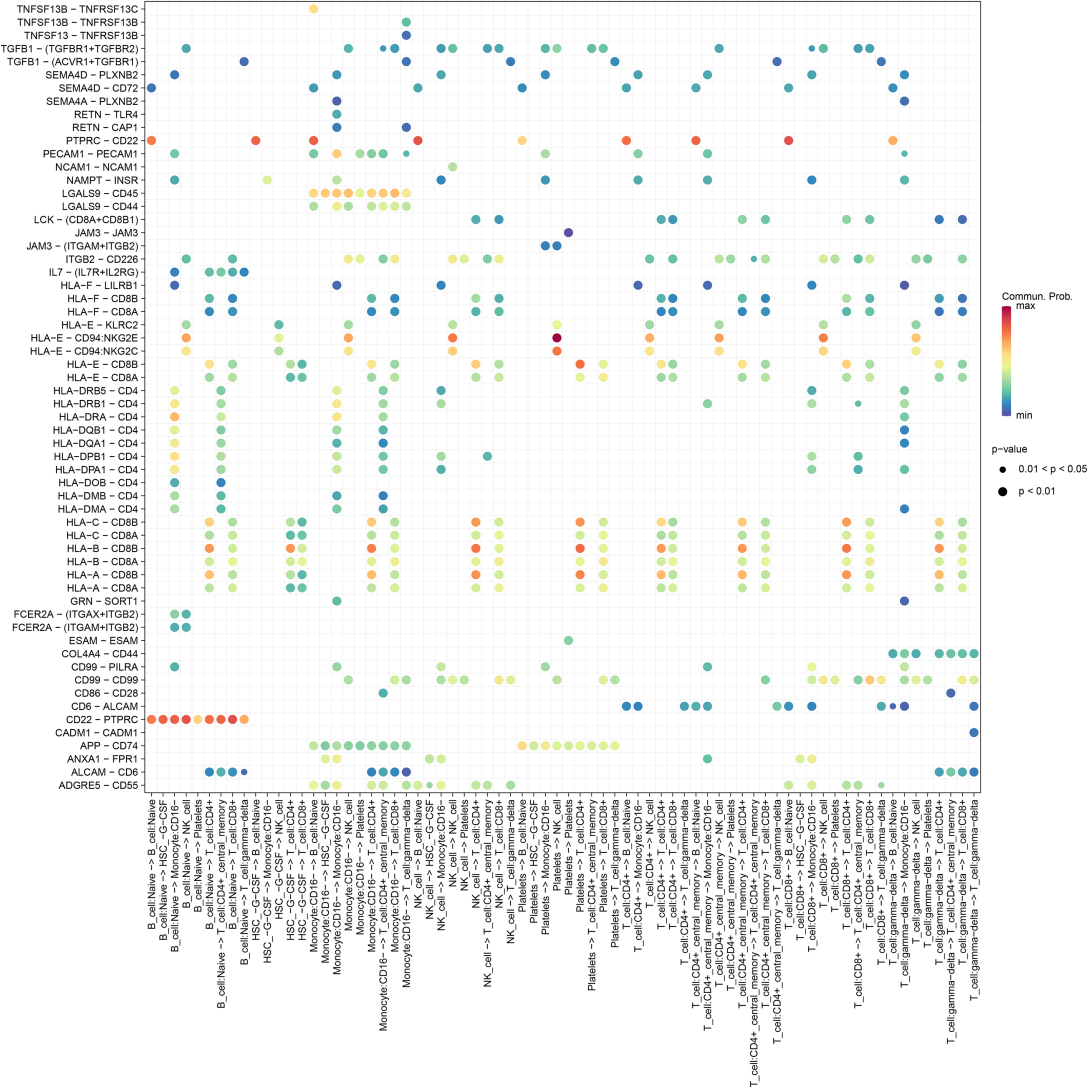
Extracted ligand–receptor pairs illustrating the relative contribution of each cell group to outgoing or incoming signals.

We used CellChat to infer the ligand–receptor signaling changes between cell types in CP *versus* control PBMCs. The analysis revealed a dense communication network with substantially elevated interactions involving naive B cells and CD8^+^ T cells in the CP samples (**Figure 6**). The key ligand–receptor pairs enriched for naive B-cell outgoing or incoming signals included CD22–PTPRC, HLA-B–CD8B, HLA-DRA–CD4, PTPRC–CD22, and ALCAM–CD6. Conversely, CD8^+^ T cells showed enhanced signaling to and from the CD16^−^ monocytes and CD4^+^ T-cell subsets, with notable pairs such as HLA-A/B–CD8B, ANXA1–FPR1, ADGRE5–CD55, and CD99–PILRA (**Figure 7**). Collectively, these inferred interactions point to specific adaptive–innate communication axes that may mediate immune dysregulation in CP.

### Cell type-specific expression of hub genes underscores candidate mechanisms in CP

**Figure 8:**
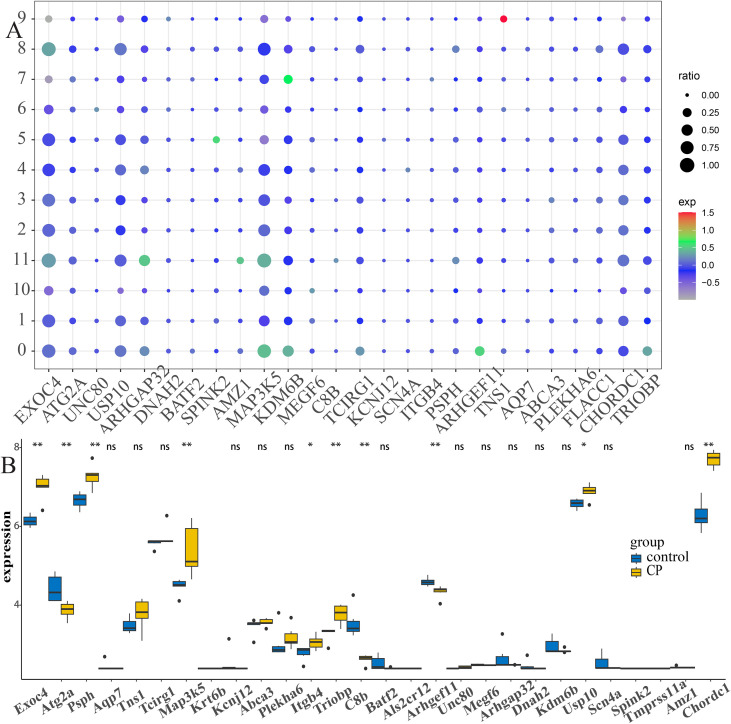
Hub gene expression in the different cell types revealing distinct patterns of chronic pancreatitis (CP). **(A)** Bubble map of the hub gene expression in the different cell types. **(B)** Box plots illustrating the mRNA expression levels of the hub genes between CP and normal controls in the public database.

The cell type-specific expression of the hub genes underscores candidate mechanisms in CP. Mapping of the 29 candidate genes across cell types revealed distinct expression patterns that align with plausible pathogenic roles. For example, CD16^−^ monocytes (clusters 0 and 9) preferentially expressed *TNS1*, *ARHGEF11*, and *MAP3K5*; CD4^+^ central memory T cells (cluster 1) enriched for *EXOC4*, *MAP3K5*, and *KDM6B*; CD8^+^ T cells (cluster 3) enriched for *EXOC4*, *MAP3K5*, and *USP10*; naive B cells (cluster 4) enriched for *EXOC4*, *KDM6B*, and *MAP3K5*; and platelets (cluster 10) enriched for *MEGF6*, *KDM6B*, and *PSPH* (**Figures 8A, B**). Differential expression analysis *versus* the healthy controls identified consistent patterns: *ATG2A*, *C8B*, and *ARHGEF11* were significantly downregulated in CP, while *EXOC4*, *PSPH*, *MAP3K5*, *ITGB4*, *TRIOBP*, *USP10*, and *CHORDC1* were upregulated. These cell type-resolved expression signatures nominate *ATG2A*, *ARHGEF11*, *EXOC4*, and several others as priority biomarkers and candidates for mechanistic follow-up.

### Screening potential diagnostic biomarkers for AP/SAP

**Figure 9:**
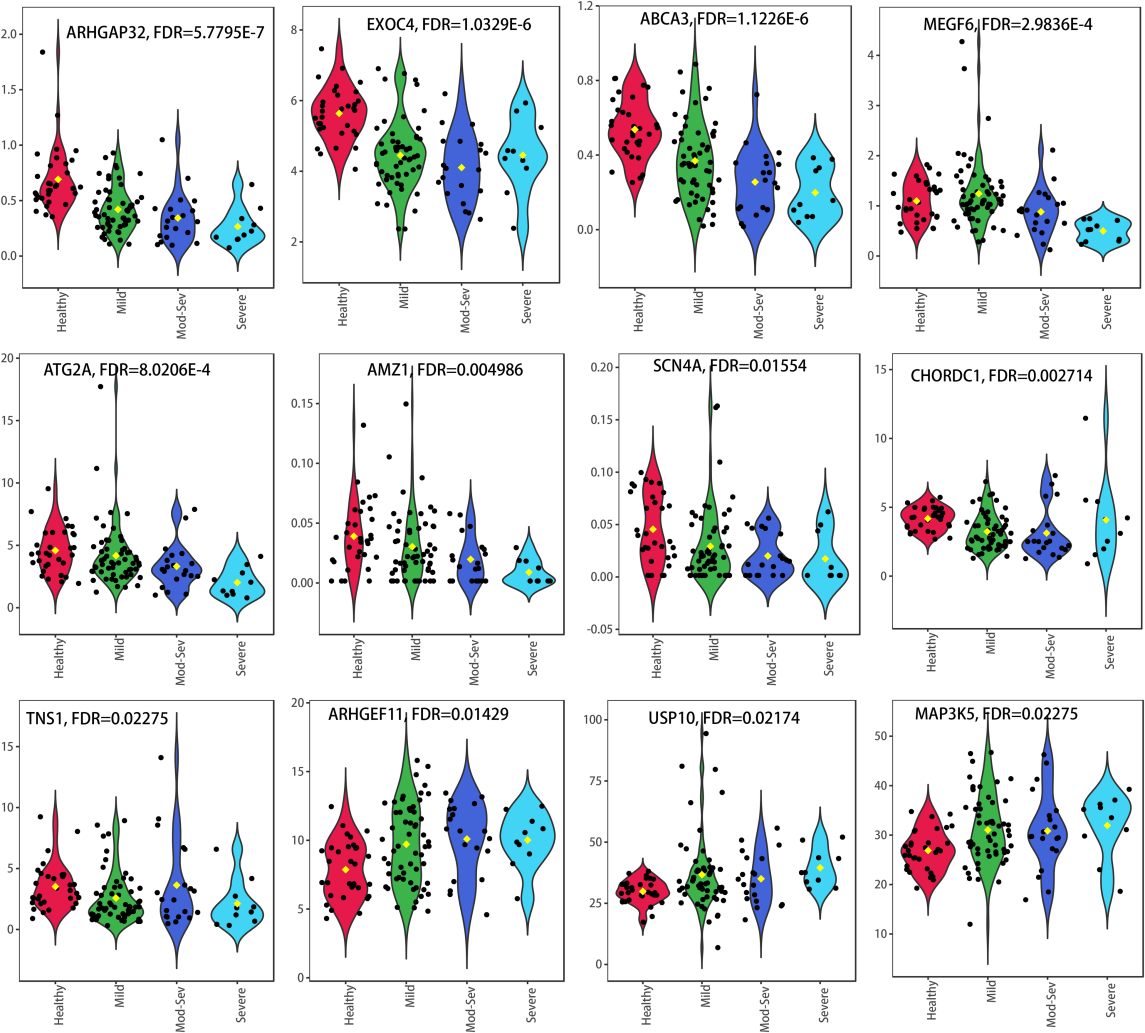
Expression patterns of the 12 differentially expressed chronic pancreatitis (CP) family-related genes (GTCPFs) across different severity levels of the pancreatitis.

Based on the 29 GTCPF genes identified from familial CP and the scRNA-seq analyses, we extracted their expression profiles in the GSE194331 AP cohort and compared the expression differences across the severity groups using one-way ANOVA with FDR correction. A total of 12 genes (i.e., *ARHGAP32*, *EXOC4*, *ABCA3*, *MEGF6*, *ATG2A*, *AMZ1*, *SCN4A*, *CHORDC1*, *TNS1*, *ARHGEF11*, *USP10*, and *MAP3K5*) met the threshold of FDR < 0.05 and displayed directionally consistent changes across the clinical spectrum from mild to severe AP (**Figure 9**). These severity-associated trajectories indicate that these GTCPF genes may serve as promising diagnostic or stratification biomarkers for AP.

### Correlation analysis between the GTCPF markers and infiltrating cells

**Figure 10:**
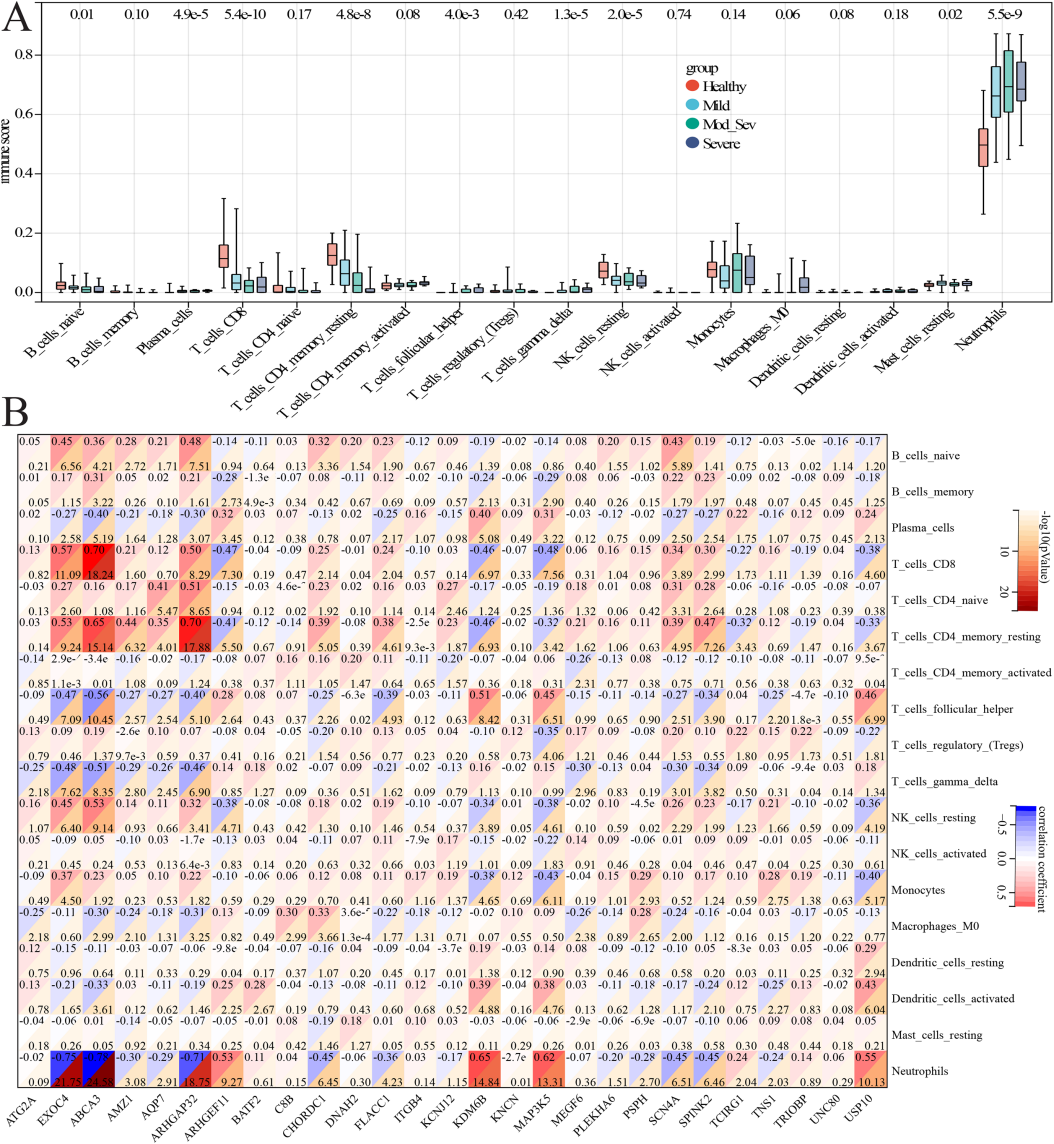
Correlation analysis of the chronic pancreatitis (CP) family-related genes (GTCPFs) and infiltrated immune cells in pancreatitis. **(A)** Box plots illustrating the expression levels of the infiltrated immune cells between acute pancreatitis (AP) and normal controls. **(B)** Correlation analysis of the GTCPFs and infiltrated immune cells during the progression of pancreatitis. The nodes in the figure are color-coded to indicate the strength and direction of correlation. Specifically, a deeper red hue signifies a more robust positive correlation, while a deeper blue hue indicates a stronger negative correlation.

Using CIBERSORT-derived immune deconvolution, we observed significant differences in the immune cell abundances across the AP severity categories (naive B cells, plasma cells, CD8^+^ T cells, resting CD4^+^ memory T cells, follicular helper T cells, γδ T cells, resting NK cells, resting mast cells, and neutrophils; *p* < 0.05) (**Figure 10A**). The correlation analysis revealed robust associations between the GTCPF expression and the immune cell levels: for instance, naive B cells correlated with *ARHGAP32* (*r* = 0.48), plasma cells inversely correlated with *ABCA3* (*r* = −0.40), CD8^+^ T cells correlated positively with *ABCA3* (*r* = 0.70), follicular helper T cells inversely correlated with *ABCA3* (*r* = −0.56), and neutrophils showed a strong negative correlation with *ABCA3* (*r* = −0.78). These relationships implicate specific GTCPF genes in the modulation of the adaptive and innate immune infiltration during disease progression (**Figure 10B**).

### WGCNA identifies GTCPF-associated co-expression modules linked to AP severity

**Figure 11:**
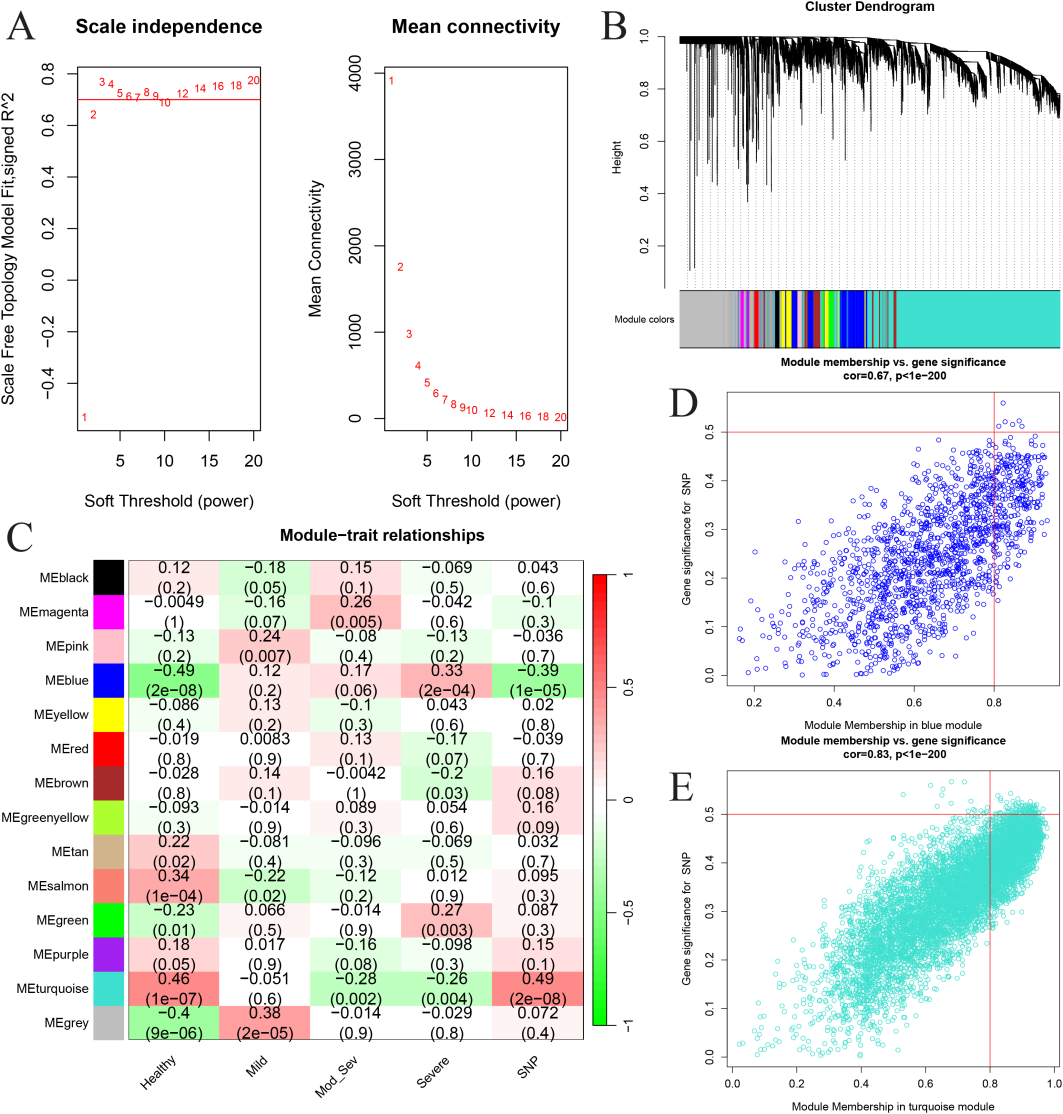
Identification of the key modules and genes related to chronic pancreatitis (CP) family-related gene (GTCPF) expression patterns and the pathogenesis of acute pancreatitis (AP). **(A)** Scale-free fit index of the different soft-threshold power and mean connectivity of various soft-threshold powers. **(B)** Cluster dendrogram plot. Different colors represent different modules. **(C)** Heat map of the association between ME modules and GTCPF gene expression patterns and the pathogenesis of AP. **(D, E)** Scatter plot of key gene screening in the blue **(D)** and turquoise **(E)** modules.

We applied WGCNA to the whole-transcriptome matrix from GSE194331 (57 mild, 20 moderately severe, 10 severe AP, and 32 controls) and selected a soft-threshold power *β* = 12 to construct a scale-free network (**Figures 11A, B**). There were 14 co-expression modules detected (**Figure 11C**). Module–trait correlation identified MEblue (*r* = −0.39, *p* = 1*e*−05) and MEturquoise (*r* = 0.49, *p* = 2*e*−08) as the top modules associated with GTCPF activity. Notably, MEblue showed an increasing positive correlation with disease severity (mild = 0.12, mod_sev = 0.17, severe = 0.33), indicating progressive module activation in severe AP. Filtering for module gene significance (GS > 0.4) and module membership (MM > 0.8) retained 97 genes in the blue module and 13 genes in the turquoise module, yielding a consolidated set of 110 putative GTCPF-related genes for downstream analyses (**Figures 11D, E**).

### Functional enrichment and network architecture of the 110 GTCPF-related genes

**Figure 12:**
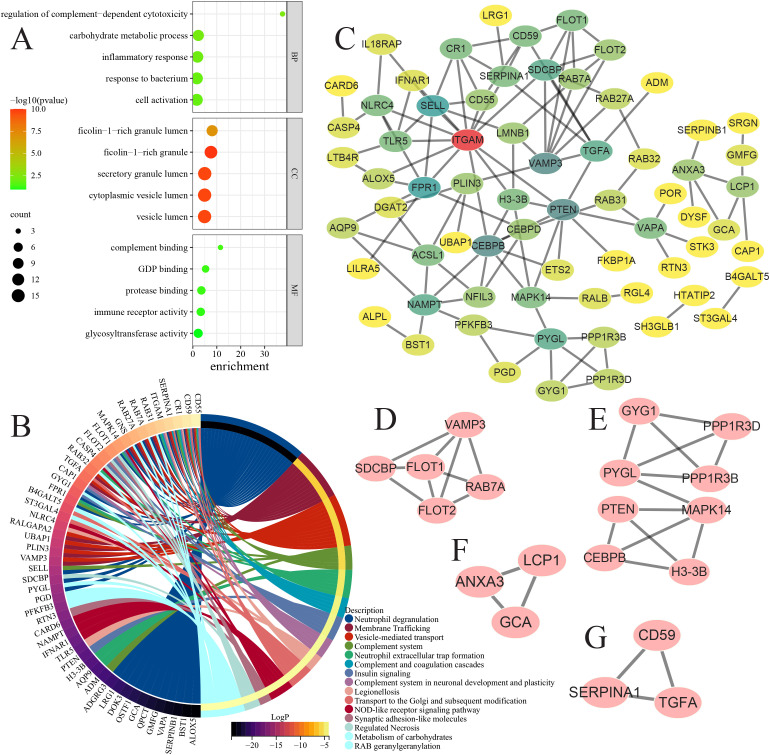
Significantly enriched functions and protein–protein interaction (PPI) network of the 110 chronic pancreatitis family (GTCPF)-related genes. **(A–C)** Significantly enriched biological processes, cellular components, and molecular functions **(A)** and pathways **(B)**. **(C)** PPI network of the 110 GTCPF-related genes. **(D–G)** PPI network of four key components identified based on MCODE.

GO and pathway enrichment of the 110 genes revealed an overrepresentation of the processes related to complement-dependent cytotoxicity regulation, carbohydrate metabolism and inflammatory responses, and localization to ficolin-1-rich and secretory granule lumens (**Figure 12A**). The pathway-level results emphasized neutrophil degranulation, membrane trafficking, and vesicle-mediated transport, with hub nodes including CD55, CD59, CR1, SERPINA1, and ITGAM (**Figure 12B**). PPI mapping followed by MCODE decomposition identified four densely connected components (MCODE score ≥ 3), suggesting that the 110 genes form several functionally coherent sub-networks that may drive the pathophysiology of AP (**Figures 12C–G**).

### Integrated development of an AP consensus signature using machine learning

**Figure 13:**
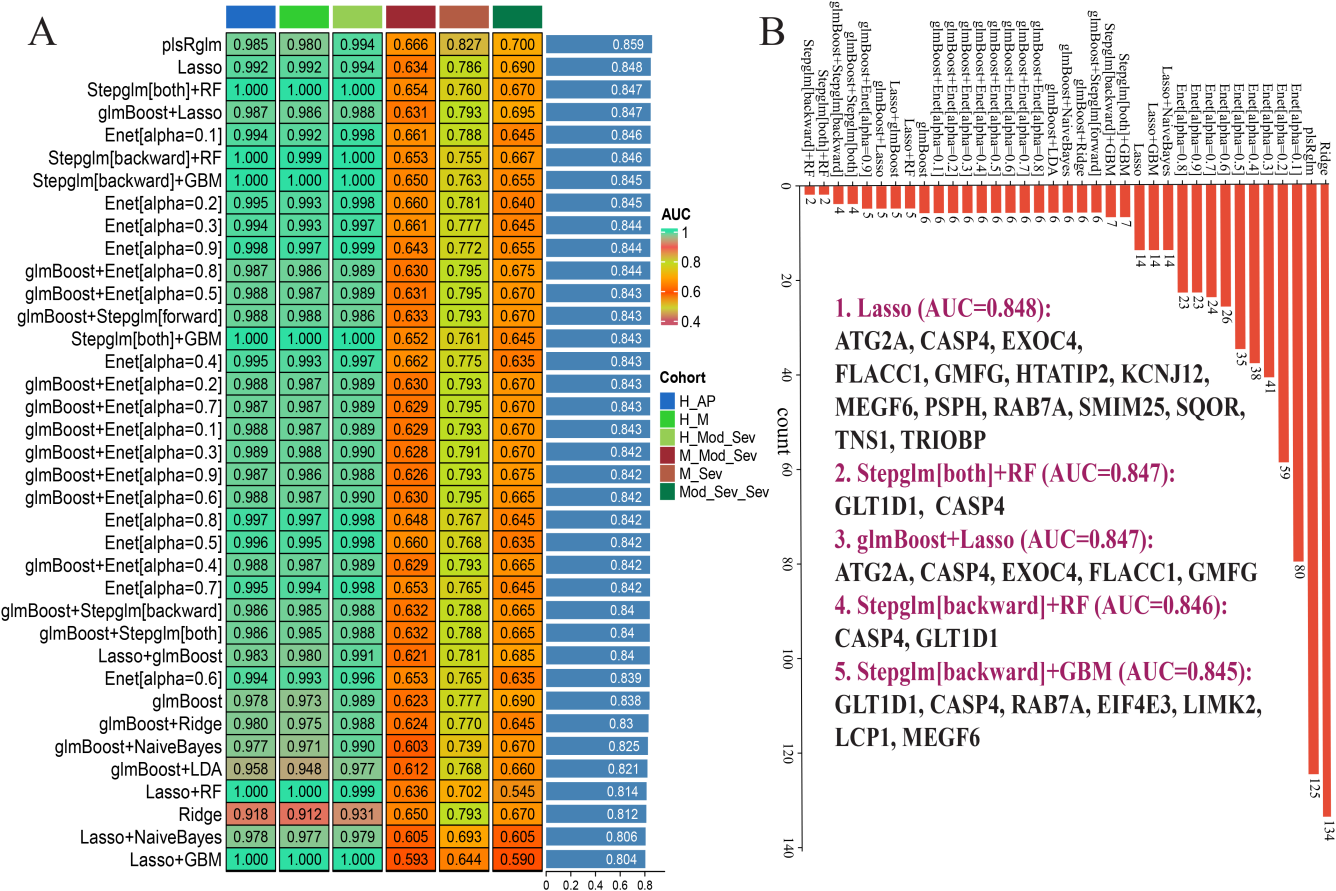
Development of an artificial intelligence-based chronic pancreatitis family (GTCPF)-related gene signature for pancreatitis. **(A)***C*-indices of 113 machine learning algorithms in acute pancreatitis (average AUC > 0.8). **(B)** Gene counts of the different algorithms.

To translate the 110-gene set into a predictive framework, we evaluated 113 algorithmic permutations spanning 10 ML families (including LASSO, CoxBoost, GBM, RF, glmBoost, Stepwise GLM) using 10-fold cross-validation in the training cohort and validation across five testing comparisons (**Figure 13**). Applying the selection criteria of average AUC > 0.80 and model parsimony (genes <20), we identified five top-performing algorithmic combinations. The LASSO model achieved the highest average AUC (0.848) using 17 genes (with *ATG2A*, *CASP4*, *EXOC4*, *FLACC1*, *GMFG*, *HTATIP2*, *KCNJ12*, *MEGF6*, *PSPH*, *RAB7A*, *SMIM25*, *SQOR*, *TNS1*, and *TRIOBP* among them). Additional high-performing combinations (e.g., Stepglm[both] + RF; glmBoost + LASSO; Stepglm[backward] + RF; and Stepglm[backward] + GBM) produced comparable AUCs (0.845–0.847) with compact gene sets, underscoring the feasibility of deriving robust, low-dimensional signatures for AP severity prediction from GTCPF-derived features. Prospective validation is required to confirm clinical utility.

## Discussion

In this study, we applied an integrated genomic and single-cell transcriptomic strategy to elucidate the genetic predisposition and immune remodeling in pancreatitis, combining familial WES, PBMC scRNA-seq, and bulk transcriptome analyses from AP cohorts. Our multilayered analysis yielded three main insights: 1) a set of novel heterozygous candidate genes segregating with familial CP (e.g., *EXOC4*, *ATG2A*, and *UNC80*) that implicate pathways such as autophagy and membrane trafficking; 2) immune reprogramming in CP and AP characterized by naive B-cell expansion and altered CD8^+^ T-cell states in CP and increased CD8^+^ T-cell abundance in severe AP; and 3) a GTCPF-derived 110-gene network that is functionally enriched for complement activity, neutrophil degranulation, and vesicle trafficking and that supports the construction of accurate ML-based severity classifiers.

Our results are based on integrative, computational analyses of sequencing and transcriptomic data; therefore, they identify associations and mechanistic hypotheses rather than establish definitive causal relationships. The identification of 12 heterozygous genes segregating in the affected family, many of which have not been previously associated with pancreatitis, suggests underappreciated genetic contributors to early-onset CP, but must be interpreted with caution given the single-pedigree design and the limited family sample size. Notably, genes such as *ATG2A* (autophagy) and *EXOC4* (exocyst/membrane trafficking) provide mechanistic hypotheses linking impaired intracellular degradation/trafficking to pancreatic injury (38). These candidate genes warrant follow-up functional validation (e.g., *in vitro* autophagy assays, CRISPR perturbation, or relevant animal models) to confirm pathogenicity and define the mechanistic pathways.

Our single-cell data revealed immune alterations that are consistent with an adaptive–innate cross-talk during CP: naive B-cell enrichment and altered T-cell differentiation states may reflect chronic antigenic stimulation or skewed immune maturation. The CellChat-inferred ligand–receptor pairs highlight putative communication axes (e.g., HLA–CD8B, CD22–PTPRC, and ANXA1–FPR1) that could mediate pathogenic inflammation or immune regulation in pancreatitis. Targeting these interactions (e.g., modulating the B-cell–T-cell communication or neutrophil degranulation) could represent therapeutic avenues. Recent studies have highlighted the importance of immune mechanisms in pancreatic pathology, emphasizing that a better understanding of these interactions could lead to innovative immunotherapeutic strategies (12, 39). Thus, further investigation into the role of these immune cell populations could provide insights into both the diagnosis and treatment of CP.

Our analysis demonstrated a notable enrichment of genes linked to crucial biological processes, including autophagy and cell adhesion, which are essential constituents of the signaling pathways implicated in CP. The identified pathways are involved in the regulation of inflammation and tissue remodeling, processes that are fundamental to the pathophysiology of CP. Accumulating evidence indicates that targeting these pathways may yield therapeutic advantages. For example, the modulation of autophagy has been put forward as a strategy to alleviate pancreatic inflammation and fibrogenesis (40). Furthermore, understanding the interactions among these pathways may uncover intricate regulatory networks that govern the cellular responses of the pancreas to injury. Through the delineation of the molecular mechanisms underlying CP, our findings contribute to the formulation of novel therapeutic approaches that target the disruption of these pathological signaling cascades, offering prospects for enhanced management strategies for patients affected by this condition.

Gaining an in-depth understanding of the interaction between AP and CP is of great significance, especially considering the role of the immune microenvironment in the progression of AP. Previous research has identified genetic factors related to pancreatitis; however, comprehensive analyses of the immune cell infiltration and the gene expression patterns at the single-cell level were not conducted (41). Through the integration of diverse methodologies, this study not only identified specific biomarkers of AP but also clarified their associations with immune cell dynamics and disease severity. These findings diverge from previous research, which predominantly concentrated on more general genetic and immunological parameters without exploring the subtle interactions at the cellular level (42). This innovative approach deepens our comprehension of the pathophysiology of AP and establishes a framework for subsequent investigations regarding targeted therapeutic strategies.

The implications of our findings for clinical practice and policymaking are significant. The identification of GTCPF-related biomarkers, such as *ARHGAP32* and *ABCA3*, which are correlated with immune cell infiltration and disease severity, indicates potential approaches for early diagnosis and personalized treatment options for patients with AP. Specifically, the insights obtained with regard to the immune microenvironment may contribute to the development of novel immunotherapeutic strategies for the management of AP and potentially chronic CP (43). Furthermore, the analysis of the signaling pathways enriched by the identified GTCPF-related genes provides crucial insights into the biological processes associated with AP. The notable enrichment of the genes related to complement-dependent cytotoxicity, carbohydrate metabolism, and inflammatory responses indicates the multifaceted characteristics of the immune response during AP. These pathways may play a key role in understanding the dysregulation of the immune functions observed in patients with AP. For example, the function of neutrophil degranulation and vesicle-mediated transport pathways implies that targeting these processes may potentially alleviate the inflammatory responses and offer therapeutic approaches for the management of AP (10, 43). Furthermore, the robust predictive model derived from the comprehensive bioinformatics analysis highlights the viability of utilizing ML methodologies to improve diagnostic precision and therapeutic effectiveness in clinical contexts (9). These advancements are promising in enhancing patient outcomes through enabling timely interventions and customizing therapies according to individual patient profiles.

Despite these promising insights, our study has several limitations that warrant consideration: i) the single-pedigree WES limits population-level inference and effect size estimation; ii) the scRNA-seq component included only a small number of PBMC samples, limiting the power to detect rare cell states and increasing the susceptibility to inter-individual variability; iii) bulk cohort analyses, despite batch correction, may retain residual heterogeneity due to the diverse collection protocols and clinical covariates; iv) environmental and lifestyle factors (i.e., diet, medication, and comorbidities) were incompletely captured in the available cohorts and may contribute to variations in the immune composition and gene expression; and v) computationally inferred interactions and modules should be considered hypothesis-generating until validated by orthogonal protein-level and functional assays.

In summary, via a thorough analysis of genomic and transcriptomic data, this research deepens the comprehension of the genetic mechanisms and immune alterations in CP. It identifies crucial genes and immune dynamics as potential biomarkers and therapeutic targets, thereby providing support for personalized medicine. Moreover, it emphasizes the role of the GTCPF-related genes in AP and the influence of the immune microenvironment on disease progression, which lays the foundation for early diagnosis and targeted therapies.

## Data Availability

The data presented in this study are deposited in the Genome Sequence Archive for Human (GSA-Human), accession number HRA015133. Due to ethical and privacy restrictions, the data are available under controlled access. Metadata are publicly accessible, and qualified researchers can apply for access to the raw data through the GSA-Human request system (https://ngdc.cncb.ac.cn/gsa-human).

## References

[1] Hines OJ Pandol SJ . Management of chronic pancreatitis. BMJ. (2024) 384:e070920. doi: 10.1136/bmj-2023-070920, PMID: 38408777

[2] Trikudanathan G Yazici C Evans Phillips A Forsmark CE . Diagnosis and management of acute pancreatitis. Gastroenterology. (2024) 167:673–88. doi: 10.1053/j.gastro.2024.02.052, PMID: 38759844

[3] Masson E Berthet S Le Gac G Le Rhun M Ka C Autret S . Identification of protease-sensitive but not misfolding PNLIP variants in familial and hereditary pancreatitis. Pancreatology. (2023) 23:507–11. doi: 10.1016/j.pan.2023.05.011, PMID: 37270400

[4] Freeman AJ Ng K Wang F Abu-El-Haija MA Chugh A Cress GA . Pancreatic enzyme use reduces pancreatitis frequency in children with acute recurrent or chronic pancreatitis: A report from INSPPIRE. Am J Gastroenterol. (2024) 119:2094–102. doi: 10.14309/ajg.0000000000002772, PMID: 38517077 PMC11452285

[5] Wei Y Wang J Qu R Zhang W Tan Y Sha Y . Genetic mechanisms of fertilization failure and early embryonic arrest: a comprehensive review. Hum Reprod Update. (2024) 30:48–80. doi: 10.1093/humupd/dmad026, PMID: 37758324

[6] Wei W Guo T Fan W Ji M Fu Y Lian C . Integrative analysis of metabolome and transcriptome provides new insights into functional components of Lilii Bulbus. Chin Herb Med. (2024) 16:435–48. doi: 10.1016/j.chmed.2023.10.004, PMID: 39072198 PMC11283230

[7] Wang Q Zhao Y Qin X Tian J . Deciphering relationship between depression and microbial molecules based on multi-omics: A case study of Chaigui Granules. Chin Herbal Medicines. (2024) 16:612–21. doi: 10.1016/j.chmed.2023.12.003, PMID: 39606256 PMC11589482

[8] Chen C Wang J Pan D Wang X Xu Y Yan J . Applications of multi-omics analysis in human diseases. MedComm (2020). (2023) 4:e315. doi: 10.1002/mco2.315, PMID: 37533767 PMC10390758

[9] Guo K Zhao Y Cao Y Li Y Yang M Tian Y . Exploring the key genetic association between chronic pancreatitis and pancreatic ductal adenocarcinoma through integrated bioinformatics. Front Genet. (2023) 14:1115660. doi: 10.3389/fgene.2023.1115660, PMID: 37501719 PMC10369079

[10] Xiao S Han X Bai S Chen R . Analysis of immune cell infiltration characteristics in severe acute pancreatitis through integrated bioinformatics. Sci Rep. (2024) 14:8711. doi: 10.1038/s41598-024-59205-1, PMID: 38622245 PMC11018854

[11] Peng C Tu G Yu L Wu P Zhang X Li Z . Murine chronic pancreatitis model induced by partial ligation of the pancreatic duct encapsulates the profile of macrophage in human chronic pancreatitis. Front Immunol. (2022) 13:840887. doi: 10.3389/fimmu.2022.840887, PMID: 35432336 PMC9011002

[12] Lin Y Chen Y Feng W Zhang J Hua R Yin B . STAT5 promotes chronic pancreatitis by enhancing GM-CSF-dependent neutrophil augmentation. J Leukoc Biol. (2021) 110:293–300. doi: 10.1002/JLB.3MA1020-647R, PMID: 34184320

[13] Hughes R Snook AE Mueller AC . The poorly immunogenic tumor microenvironment of pancreatic cancer: the impact of radiation therapy, and strategies targeting resistance. Immunotherapy. (2022) 14:1393–405. doi: 10.2217/imt-2022-0046, PMID: 36468417

[14] Chen S Zhou Y Chen Y Gu J . fastp: an ultra-fast all-in-one FASTQ preprocessor. Bioinformatics. (2018) 34:i884–90. doi: 10.1093/bioinformatics/bty560, PMID: 30423086 PMC6129281

[15] Li H Durbin R . Fast and accurate long-read alignment with Burrows-Wheeler transform. Bioinformatics. (2010) 26:589–95. doi: 10.1093/bioinformatics/btp698, PMID: 20080505 PMC2828108

[16] Li H Handsaker B Wysoker A Fennell T Ruan J Homer N . The sequence alignment/map format and SAMtools. Bioinformatics. (2009) 25:2078–9. doi: 10.1093/bioinformatics/btp352, PMID: 19505943 PMC2723002

[17] McKenna A Hanna M Banks E Sivachenko A Cibulskis K Kernytsky A . The Genome Analysis Toolkit: a MapReduce framework for analyzing next-generation DNA sequencing data. Genome Res. (2010) 20:1297–303. doi: 10.1101/gr.107524.110, PMID: 20644199 PMC2928508

[18] Wang K Li M Hakonarson H . ANNOVAR: functional annotation of genetic variants from high-throughput sequencing data. Nucleic Acids Res. (2010) 38:e164. doi: 10.1093/nar/gkq603, PMID: 20601685 PMC2938201

[19] Talevich E Shain AH Botton T Bastian BC . CNVkit: genome-wide copy number detection and visualization from targeted DNA sequencing. PLoS Comput Biol. (2016) 12:e1004873. doi: 10.1371/journal.pcbi.1004873, PMID: 27100738 PMC4839673

[20] Layer RM Chiang C Quinlan AR Hall IM . LUMPY: a probabilistic framework for structural variant discovery. Genome Biol. (2014) 15:R84. doi: 10.1186/gb-2014-15-6-r84, PMID: 24970577 PMC4197822

[21] Vaser R Adusumalli S Leng SN Sikic M Ng PC . SIFT missense predictions for genomes. Nat Protoc. (2016) 11:1–9. doi: 10.1038/nprot.2015.123, PMID: 26633127

[22] Adzhubei I Jordan DM Sunyaev SR . Predicting functional effect of human missense mutations using PolyPhen-2. Curr Protoc Hum Genet. (2013). Chapter 7: p. Unit7.20. doi: 10.1002/0471142905.hg0720s76, PMID: 23315928 PMC4480630

[23] Chun S Fay JC . Identification of deleterious mutations within three human genomes. Genome Res. (2009) 19:1553–61. doi: 10.1101/gr.092619.109, PMID: 19602639 PMC2752137

[24] Reva B Antipin Y Sander C . Determinants of protein function revealed by combinatorial entropy optimization. Genome Biol. (2007) 8:R232. doi: 10.1186/gb-2007-8-11-r232, PMID: 17976239 PMC2258190

[25] Reva B Antipin Y Sander C . Predicting the functional impact of protein mutations: application to cancer genomics. Nucleic Acids Res. (2011) 39:e118. doi: 10.1093/nar/gkr407, PMID: 21727090 PMC3177186

[26] Shihab HA Gough J Cooper DN Day IN Gaunt TR . Predicting the functional consequences of cancer-associated amino acid substitutions. Bioinformatics. (2013) 29:1504–10. doi: 10.1093/bioinformatics/btt182, PMID: 23620363 PMC3673218

[27] Shihab HA Rogers MF Gough J Mort M Cooper DN Day IN . An integrative approach to predicting the functional effects of non-coding and coding sequence variation. Bioinformatics. (2015) 31:1536–43. doi: 10.1093/bioinformatics/btv009, PMID: 25583119 PMC4426838

[28] Choi Y Sims GE Murphy S Miller JR Chan AP . Predicting the functional effect of amino acid substitutions and indels. PLoS One. (2012) 7:e46688. doi: 10.1371/journal.pone.0046688, PMID: 23056405 PMC3466303

[29] Kim S Jhong JH Lee J Koo JY . Meta-analytic support vector machine for integrating multiple omics data. BioData Min. (2017) 10:2. doi: 10.1186/s13040-017-0126-8, PMID: 28149325 PMC5270233

[30] Kircher M Witten DM Jain P O'Roak BJ Cooper GM Shendure J . A general framework for estimating the relative pathogenicity of human genetic variants. Nat Genet. (2014) 46:310–5. doi: 10.1038/ng.2892, PMID: 24487276 PMC3992975

[31] Zhou Y Zhou B Pache L Chang M Khodabakhshi AH Tanaseichuk O . Metascape provides a biologist-oriented resource for the analysis of systems-level datasets. Nat Commun. (2019) 10:1523. doi: 10.1038/s41467-019-09234-6, PMID: 30944313 PMC6447622

[32] Bader GD Hogue CW . An automated method for finding molecular complexes in large protein interaction networks. BMC Bioinf. (2003) 4:2. doi: 10.1186/1471-2105-4-2, PMID: 12525261 PMC149346

[33] Macosko EZ Basu A Satija R Nemesh J Shekhar K Goldman M . Highly parallel genome-wide expression profiling of individual cells using nanoliter droplets. Cell. (2015) 161:1202–14. doi: 10.1016/j.cell.2015.05.002, PMID: 26000488 PMC4481139

[34] Klein AM Mazutis L Akartuna I Tallapragada N Veres A Li V . Droplet barcoding for single-cell transcriptomics applied to embryonic stem cells. Cell. (2015) 161:1187–201. doi: 10.1016/j.cell.2015.04.044, PMID: 26000487 PMC4441768

[35] Korsunsky I Millard N Fan J Slowikowski K Zhang F Wei K . Fast, sensitive and accurate integration of single-cell data with Harmony. Nat Methods. (2019) 16:1289–96. doi: 10.1038/s41592-019-0619-0, PMID: 31740819 PMC6884693

[36] Gulati GS Sikandar SS Wesche DJ Manjunath A Bharadwaj A Berger MJ . Single-cell transcriptional diversity is a hallmark of developmental potential. Science. (2020) 367:405–11. doi: 10.1126/science.aax0249, PMID: 31974247 PMC7694873

[37] Shen W Song Z Zhong X Huang M Shen D Gao P . Sangerbox: A comprehensive, interaction-friendly clinical bioinformatics analysis platform. Imeta. (2022) 1:e36. doi: 10.1002/imt2.36, PMID: 38868713 PMC10989974

[38] Khan E Chakrabarty S Shariff S Bardhan M . Genetics and genomics of chronic pancreatitis with a focus on disease biology and molecular pathogenesis. Glob Med Genet. (2023) 10:324–34. doi: 10.1055/s-0043-1776981, PMID: 38025192 PMC10665123

[39] Gitto SB Beardsley JM Nakkina SP Oyer JL Cline KA Litherland SA . Identification of a novel IL-5 signaling pathway in chronic pancreatitis and crosstalk with pancreatic tumor cells. Cell Commun Signal. (2020) 18:95. doi: 10.1186/s12964-020-00594-x, PMID: 32552827 PMC7302008

[40] Glaubitz J Asgarbeik S Lange R Mazloum H Elsheikh H Weiss FU . Immune response mechanisms in acute and chronic pancreatitis: strategies for therapeutic intervention. Front Immunol. (2023) 14:1279539. doi: 10.3389/fimmu.2023.1279539, PMID: 37881430 PMC10595029

[41] Fang Z Li J Cao F Li F . Integration of scRNA-seq and bulk RNA-seq reveals molecular characterization of the immune microenvironment in acute pancreatitis. Biomolecules. (2022) 13:78. doi: 10.3390/biom13010078, PMID: 36671463 PMC9855877

[42] Li G Chen H Liu L Xiao P Xie Y Geng X . Role of interleukin-17 in acute pancreatitis. Front Immunol. (2021) 12:674803. doi: 10.3389/fimmu.2021.674803, PMID: 34594321 PMC8476864

[43] Gu X Huang Z Ying X Liu X Ruan K Hua S . Ferroptosis exacerbates hyperlipidemic acute pancreatitis by enhancing lipid peroxidation and modulating the immune microenvironment. Cell Death Discov. (2024) 10:242. doi: 10.1038/s41420-024-02007-1, PMID: 38773098 PMC11109150

